# Spinal Mechanisms of Pain Modulation by Spinal Cord Stimulation: A Systematic Review

**DOI:** 10.7759/cureus.85567

**Published:** 2025-06-08

**Authors:** Jaden Y Fang, Hideaki Yamamoto, Adam Romman, Aristides P Koutrouvelis, Satoshi Yamamoto

**Affiliations:** 1 Anesthesiology, University of Texas Medical Branch, Galveston, USA; 2 Biological Sciences, University of California San Diego, San Diego, USA

**Keywords:** pain mitigation, scs mechanism, spinal cord-level mechanisms, spinal cord stimulation (scs), systematic literature review

## Abstract

Spinal cord stimulation (SCS) is a widely used neuromodulation therapy for chronic neuropathic pain, including failed back surgery syndrome and complex regional pain syndrome, but its mechanisms of action remain incompletely defined. This systematic review examined 40 unique preclinical animal studies to classify spinal mechanisms underlying SCS-induced analgesia. A comprehensive database search including PubMed, MEDLINE, and Cochrane was conducted through October 2024 following PRISMA guidelines. Studies were included if they investigated SCS effects on spinal cord cells such as dorsal horn neurons, dorsal column fibers, interneurons, or glia, and excluded if they involved brain structures. Mechanisms were categorized into three domains: inhibition of ascending nociceptive transmission (n = 22), enhancement of descending inhibition (n = 5), and neuroimmune modulation via microglial and astrocytic pathways (n = 13). SCS was shown to enhance inhibitory signaling, reduce excitatory neurotransmitter release, and modulate dorsal horn activity at molecular and electroneurophysiological levels. It also promoted descending inhibition via serotonergic, opioid, and cholinergic mechanisms. Neuroimmune effects included suppression of proinflammatory cytokines and modulation of microglial and astrocyte activity, often through MAPK-related signaling. Risk of bias was assessed using the SYRCLE tool, revealing a variable methodological quality. The experimental frameworks utilized either neuropathic or inflammatory pain models, which exhibit substantial clinical relevance to chronic pain phenomena. Collectively, these findings suggest that SCS exerts analgesic effects through integrated spinal mechanisms involving neuronal inhibition, descending modulation, and glial suppression. However, the exclusive reliance on animal models limits direct clinical translatability, and future studies are needed to validate whether these mechanistic insights reliably extend to human physiology and therapeutic outcomes. This review provides a mechanistic framework to guide translational strategies for optimizing SCS therapy.

## Introduction and background

Spinal cord stimulation (SCS) is an established neuromodulation therapy for managing pain conditions by delivering electrical impulses to the spinal cord [1]. The procedure involves the subcutaneous implantation of a pulse generator and the placement of electrodes in the epidural space adjacent to the spinal cord. Clinically, SCS is widely used for chronic neuropathic pain, including conditions such as failed back surgery syndrome (FBSS) and complex regional pain syndrome [2-3].

Currently, the advancements in neuromodulation practices, particularly SCS, have increased in therapeutic relevance and bolstered patient forecasts [4]. Conventional SCS typically employs tonic stimulation at frequencies of 40-60 Hz [5]. This modality is thought to target A-beta fibers in the dorsal column and work via the gate control theory of pain. Approximately a decade ago, high-frequency stimulation came to market with a proposed mechanism of action of modulation of dorsal horn neurons. Promising clinical results spurred a revolution in new waveform programming. Among these innovations, burst stimulation delivers packets of high-frequency pulses (500 Hz) at rate of 40 bursts per second, designed to emulate the physiological firing patterns of endogenous neurons and enhance analgesic efficacy [6]. Other recent SCS algorithms include closed-loop systems that use feedback control at the dorsal column and “differential target multiplexed” waveform that acts on glial cells [5]. These mechanisms remain poorly understood and unproven, and a stronger mechanistic understanding has the potential to refine device development and programming to improve clinical outcomes. Animal models of neuropathic pain allow for controlled investigations into how SCS affects nervous system structures and pathways implicated in pain processing [7]. Nevertheless, the underlying mechanisms associated with analgesic properties remain inadequately understood [8].

In light of the increasing dependence on SCS within clinical paradigms and the considerable volume of preclinical investigations conducted, it is both opportune and imperative to evaluate the extent to which experimental models have contributed to our comprehension of the biological mechanisms correlated with SCS-induced pain modulation and the processes through which pain manifests or recurs, particularly in individuals afflicted with FBSS, who are most frequently representative of candidates for SCS therapy [9].

The primary objective of this systematic review is to amalgamate and classify the relevant evidence sourced from studies that probe into mechanisms underlying SCS-induced analgesia in animal research. This assessment intends to deliver a broad perspective on the mechanisms that have been reviewed until now in the domain of preclinical research and to suggest possible channels for subsequent investigation. Such insights provide a framework to inform translational initiatives aimed at enhancing the efficacy and specificity of SCS in its prospective applications.

## Review

Methods

To better understand the underlying mechanisms of SCS pain alleviation in animals, we systematically reviewed cohort studies from inception to October 18, 2024. This systematic review has been duly registered with the International Platform of Registered Systematic Review and Meta-analysis Protocols (INPLASY) (registration number: 202540047). The search flow diagram strictly follows the Preferred Reporting Items for Systematic Reviews and Meta-Analyses (PRISMA) guidelines.

Search Strategy

A comprehensive systematic search was conducted using the Cochrane Central Register of Control Trials. The search strategy is detailed in Appendix A.

Data Extraction

To guarantee the thoroughness of our search, the reference lists of sourced articles were meticulously scrutinized. Two authors (J.F. and H.Y.) independently undertook the data extraction process. Any discrepancies that arose were resolved through a consensus mechanism among the authors. Following the search, two independent reviewers evaluated the articles to ascertain their adherence to the inclusion criteria.

Study Selection

Pain is defined by the International Association for the Study of Pain (IASP) as “an unpleasant sensory and emotional experience associated with, or resembling that associated with, actual or potential tissue damage” [10]. Our search was conducted in close consultation with a professional medical librarian to ensure a comprehensive and systematic retrieval of relevant studies that described potential mechanisms of pain within the context of SCS application in animals. Following an initial broad systematic search to identify relevant literature as described in Appendix A, only studies published from inception to date and in English were included. Included experimental and computational studies described quantifiable outcome measures and explicitly defined necessary interventions between a control group and an experimental group. Studies utilizing spinal cord cells, including dorsal column cells, dorsal horn cells, glial cells, and interneurons, were included. Studies utilizing brain or periaqueductal gray cells were excluded from our study. Systematic review studies or studies without the use of animals were excluded from our search. Research comparing different frequencies of SCS in its efficacy without describing pain and mechanisms was also excluded. Finally, studies involving brain cells were excluded to ensure that only those specifically related to spinal cord cells were included. A detailed description of the full search strategy is provided in the appendices.

Risk-of-Bias Assessment

To assess the risk of bias in this analysis of animal studies, the SYRCLE Risk of Bias (RoB) tool was utilized. This tool is an adaptation of the Cochrane Collaboration Risk of Bias tool, originally designed for human studies, and is tailored to address specific challenges in preclinical animal research. The SYRCLE RoB tool comprises 10 items aimed at evaluating potential biases, including selection, performance, detection, attrition, and reporting. A response of "yes" to an item indicates a low risk of bias, while a "no" corresponds to a high risk of bias. Responses that cannot be clearly categorized are deemed to indicate an unclear risk of bias. Of note, the SYRCLE RoB tool does not recommend the use of summary scores for reported items.

Data Collection

Our principal outcome is to identify, categorize, and summarize the pain mechanism of SCS in animals. Therefore, demographics, experimental characteristics, and study conclusions were collected from our study. Selected articles were collected by author(s), publication year, species, cell type, pain model used, and conclusion. This report was composed in accordance with the PRISMA checklist.

Results

Study Identification and Inclusion

**Figure 1:**
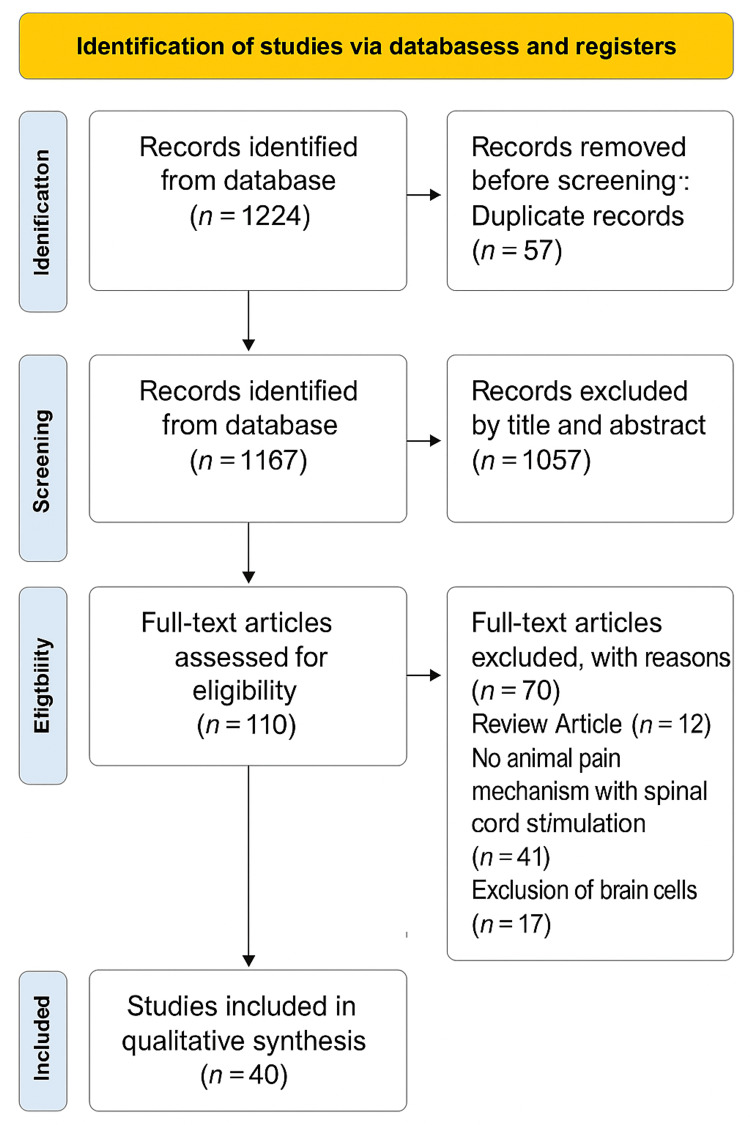
PRISMA flow diagram of the study selection process for pain mechanisms modulated by SCS in spinal cord cells of animals PRISMA, Preferred Reporting Items for Systematic Reviews and Meta-Analyses; SCS, spinal cord stimulation

In the examination of pain mechanisms modulated by SCS in spinal cord cells of animal models, an initial identification included 1,224 records. Of that, 57 duplicates were removed before screening. Of the remaining 1,167 records, 1,057 were excluded based on criteria from titles and abstracts. The residual 110 records were meticulously screened against the relevant exclusion criteria concerning systematic reviews, human subjects, brain cells, and periaqueductal gray cells. Finally, 40 full-text articles were selected for further analysis (Figure 1).

The studies included in this review are summarized in Table 1, which provides an overview of author(s), publication year, species, cell type, pain model used, and key conclusions. To enhance the clarity and depth of analysis, studies were further categorized based on their underlying mechanisms into three distinct groups: inhibition of ascending nociceptive transmission at the dorsal horn (Table 1), enhancement of descending inhibition (Table 2), and neuroimmune modulation: microglial and astrocytic effects (Table 3). Among the 40 full-text articles reviewed, 22 were classified under inhibition of ascending nociceptive transmission, 5 under descending inhibition, and 13 under neuroimmune modulation.

**Table 1:** Articles categorized with SCS's pain-relieving mechanism related to inhibition of ascending nociceptive transmission at dorsal horn CB1, cannabinoid receptor 1; c-Fos, cellular-Fos; CSF1, colony-stimulating factor 1; DC, dorsal column; DH, dorsal horn; DRG, dorsal root ganglion; ECAP, evoked compound action potential; EMG, electromyography; GABA, gamma-aminobutyric acid; GABA(A), gamma-aminobutyric acid type A receptor; GABA(B), gamma-aminobutyric acid type B receptor; GAD65, glutamate decarboxylase 65; HF-SCS, high-frequency spinal cord stimulation; KCC2, potassium-chloride cotransporter 2; SCS, spinal cord stimulation; SNL, spinal nerve ligation

Author	Species	Cell Type	Pain Model Used	Conclusion
Molecular biology study				
					Koetsier E et al., 2020 [11]	Rats	Neurons (DRG)	Neuropathic pain model	DRG stimulation does not rely on dorsal horn GABA release, indicating distinct pain relief mechanisms from conventional SCS.
Sun et al., 2017 [12]	Rats	Neurons (CB1 receptor-expressing)	Neuropathic pain model	Repetitive SCS induces long-lasting analgesia via CB1 receptor–mediated endocannabinoid activation.
Crosby et al., 2015 [13]	Rats	Neurons (GABAergic interneurons)	Neuropathic pain model	Burst and tonic SCS both alleviate pain, but only tonic SCS acts through spinal GABAergic mechanisms, while burst SCS produces similar analgesia via GABA-independent pathways.
Zhang et al., 2015 [14]	Rats	Neurons (projection neurons)	Inflammatory pain model	SCS modulates projection neuron activity via mechanisms beyond classical gate control, implicating broad GABA(A) neurotransmission.
Zhang et al., 2014 [15]	N/A	Neurons (wide-dynamic range)	Inflammatory pain model	Modeling indicates GABAergic inhibition modulates optimal SCS frequencies and the analgesic effect on wide-dynamic range neurons.
Ultenius et al., 2013 [16]	Rats	Neurons (GABAergic)	Inflammatory pain model	Enhanced local GABA synthesis in the dorsal horn (increased GAD65 in lamina II) is linked to effective SCS analgesia.
Janssen et al., 2012 [17]	Rats	Neurons (GABAergic, KCC2-expressing)	Inflammatory pain model	Impaired KCC2-dependent GABA(A) inhibition is observed in diabetic neuropathy, which may affect SCS efficacy.
Smits et al., 2009 [18]	Rats	Neurons	Neuropathic pain model	SCS increases c-Fos expression in the dorsal horn, marking both early and late neuronal activation in neuropathic pain.
Cui et al., 1998 [19]	Rats	Neurons (GABAergic and adenosine receptor-expressing)	Inflammatory pain model	Combined GABA(B) and adenosine receptor activation is crucial for SCS-induced normalization of tactile thresholds.
Cui et al., 1997 [20]	Rats	Neurons (GABAergic and excitatory)	Neuropathic pain model	SCS reduces neuropathic pain by activating GABAergic mechanisms that suppress excitatory amino acid release, reversed by GABA(B) antagonism.
Cui et al., 1996 [21]	Rats	Neurons (GABAergic in dorsal horn)	Inflammatory pain model	SCS enhances spinal GABAergic function, predominantly via GABA(B) receptors, to suppress touch-evoked allodynia.
Stiller et al., 1996 [22]	Rats	Neurons (GABAergic in dorsal horn)	Inflammatory pain model	SCS increases GABA release in the dorsal horn, correlating with suppression of tactile allodynia in neuropathic rats.
Linderoth et al., 1994 [23]	Rats	Neurons (GABAergic in dorsal horn)	Inflammatory pain model	SCS significantly increases GABA release in the dorsal horn.
Electroneurophysiological study				
					Calvert et al., 2023 [24]	Rats	Spinal dorsal horn	Neuropathic pain model	Distinct ECAP and EMG responses during lateral stimulation reveal preferential ipsilateral recruitment.
Kuo et al., 2023 [25]	Rats	Neurons	Neuropathic pain model	SCS modulates dorsal horn neurons in a frequency- and duration-dependent manner, preferentially facilitating inhibitory interneurons.
Gilbert et al., 2022 [26]	–	Neurons	Neuropathic pain model	Low-frequency subperception SCS activates few DC axons that inhibit DH neurons, producing rapid analgesia.
Song et al., 2014 [27]	Rats	Neurons	Neuropathic pain model	Both HF-SCS and conventional SCS effectively reduce hypersensitivity via primarily segmental mechanisms. Conventional SCS activates ascending pathways in the gracile nucleus while HF-SCS does not.
Yang et al., 2014 [28]	Rats	Neurons (wide-dynamic range)	Neuropathic pain model	Dorsal column SCS shows intensity-dependent inhibition of wide-dynamic range neurons, effectively reducing nociceptive transmission.
Smits et al., 2012 [29]	Rats	Neurons (dorsal column)	Neuropathic pain model	Dorsal column SCS at the entry level of injured fibers yields superior pain relief via segmental mechanisms.
Yang et al., 2011 [30]	Rats	Neurons (A-fiber afferents)	Neuropathic pain model	Spinal cord stimulation reduced mechanical hypersensitivity in SNL rats using stimulus intensities below the Aα/β threshold, indicating effective pain relief by selectively recruiting a small subset of A-fiber afferents.
Wallin et al., 2003 [31]	Rats	Neurons (wide-dynamic range)	Neuropathic pain model	SCS reduces long-term potentiation of C-fiber mediated responses, thereby modulating central sensitization.
Yakhnitsa et al., 1999 [32]	Rats	Neurons (dorsal horn)	Neuropathic pain model	SCS suppresses dorsal horn hyperexcitability, significantly reducing both principal responses and afterdischarges.

**Table 2:** Articles categorized with SCS's pain-relieving mechanism related to enhancement of descending inhibition 5-HT, 5-hydroxytryptamine; GABA, gamma-aminobutyric acid; SCS, spinal cord stimulation

Author	Species	Cell Type	Pain Model Used	Conclusion
Zhai et al., 2022 [33]	Rats	Neurons, opioid receptors	Neuropathic pain model	SCS-induced analgesia is mediated by frequency-dependent opioid peptide release, as evidenced by increased methionine enkephalin blocked by antagonists.
Sato et al., 2013 [34]	Rats	Neurons (opioid receptor-expressing)	Neuropathic pain model	SCS reduces hypersensitivity via frequency-dependent activation of opioid receptors (μ at 4 Hz, δ at 60 Hz).
Song et al., 2011 [35]	Rats	Neurons (serotonergic)	Neuropathic pain model	Activation of spinal 5-HT receptors is essential for SCS-induced analgesia, potentially via a GABAergic mechanism.
Song et al., 2009 [36]	Rats	Neurons (serotonergic)	Inflammatory pain model	Serotonergic mechanisms enhance SCS analgesia, with descending serotonergic pathways modulating pain via a GABAergic link.
Schechtmann et al., 2008 [37]	Rats	Neurons (cholinergic)	Neuropathic pain model	SCS analgesia is mediated by activation of the cholinergic system via muscarinic (M4) receptors.

**Table 3:** Articles categorized with SCS's pain-relieving mechanism related to neuroimmune modulation: microglial and astrocyte effects AFI, afferent fiber index; CSF1, colony-stimulating factor 1; DRG, dorsal root ganglion; DTMP, differential target multiplexed programming; HF10 SCS, 10-kilohertz high-frequency spinal cord stimulation; MT, motor threshold; NF-κB, nuclear factor kappa B; SCS, spinal cord stimulation; TLR4/NF-κB, toll-like receptor 4/nuclear factor kappa-light-chain-enhancer of activated B cells signaling pathway

Author	Species	Cell Type	Pain Model Used	Conclusion
Yu et al., 2024 [38]	Rats	Neurons, microglia	Neuropathic pain model	HF10 SCS inactivates the microglial Kaiso–P2X7 axis, reducing inflammation and providing long-lasting pain relief.
Cedeño et al., 2023 [39]	Rats	Neurons	Neuropathic pain model	Differential target multiplexed SCS improves mechanical sensitivity and alters neuron/glia transcriptomic profiles.
Ni et al., 2023 [40]	Rats	Neurons, microglia, astrocytes	Neuropathic pain model	SCS suppresses neuroinflammatory markers (TLR4, NF-κB, IL-1β, IL-6, TNF-α) to alleviate pain hypersensitivity.
Sun et al., 2022 [41]	Rats	Neurons, microglia	Neuropathic pain model	SCS alleviates neuropathic pain by reducing CSF1-mediated microglial activation.
Smith et al., 2021 [42]	Rats	Neurons, microglia	Neuropathic pain model	Differential SCS programming (DTMP) modulates microglial activation toward a healthier profile.
Tilley et al., 2021 [43]	Rats	Neurons, astrocytes	Neuropathic pain model	SCS induces extensive proteomic changes affecting stress, oxidation, and neuron-glial pathways.
Wang et al., 2021 [44]	Rats	Neurons, microglia	Neuropathic pain model	SCS reduces cardiac pain by inhibiting microglial activation and inflammatory cytokines via the p38 MAPK pathway.
Liao et al., 2020 [45]	Rats	Neurons, microglia	Neuropathic pain model	Early high-frequency SCS attenuates mechanical hyperalgesia by inhibiting MAPK activation in DRG and dorsal horn.
Shinoda et al., 2020 [46]	Rats	Neurons, microglia	Neuropathic pain model	SCS reduces neuropathic pain by suppressing superficial microglial activation in the L4 dorsal horn.
Vallejo et al., 2020 [47]	Rats	Neurons, astrocytes	Neuropathic pain model	Specific SCS waveforms differentially modulate behavior and gene expression associated with chronic pain.
Jongen et al., 2014 [48]	Rats	Neurons, glial cells	Neuropathic pain model	SCS rapidly reduces spinal metabolic activity (visualized by AFI), indicating its acute modulatory effect in neuropathic pain.
Sato et al., 2014 [49]	Rats	Neurons, microglia, astrocytes	Neuropathic pain model	SCS (6-hour at 90% MT) significantly reduces mechanical hyperalgesia and glial activation.
Yuan et al., 2014 [50]	Rats	Neurons and glial cells (TLR4-expressing)	Neuropathic pain model	SCS attenuates neuropathic pain by inhibiting the TLR4/NF-κB pathway and lowering pro-inflammatory cytokine levels.

To assess the risk of bias of the included studies, the SYRCLE RoB tool was utilized (Table 4). As recommended by the SYRCLE RoB tool, summary scores were not tabulated for the articles.

**Table 4:** SYRCLE’s risk-of-bias assessment for included studies

Author	Sequence Generation	Baseline Characteristics	Allocation Concealment	Random Housing	Blinding (Performance Bias)	Random Outcome Assessment	Blinding (Detection Bias)	Incomplete Outcome Data	Selective Outcome Reporting	Other Sources of Bias
Yu et al., 2024 [38]	Yes	Yes	No	No	No	Yes	Yes	Yes	No	No
Cedeño et al., 2023 [39]	No	No	No	No	Yes	Yes	No	No	Yes	No
Kuo et al., 2023 [25]	Yes	Yes	Yes	Yes	Yes	Yes	Yes	Yes	No	Yes
Calvert et al., 2023 [24]	No	No	No	No	Yes	Yes	No	No	Yes	No
Ni et al., 2023 [40]	Yes	Yes	No	Yes	Yes	Yes	No	Yes	No	Yes
Sun et al., 2022 [41]	Yes	Yes	No	Yes	Yes	No	Yes	Yes	Yes	No
Zhai et al., 2022 [33]	Yes	Yes	No	Yes	Yes	No	No	Yes	No	No
Smith et al., 2021 [42]	Yes	Yes	Yes	Yes	Yes	No	Yes	Yes	Yes	Yes
Wang et al., 2021 [44]	Yes	Yes	No	Yes	Yes	No	Yes	Yes	No	No
Tilley et al., 2021 [43]	Yes	Yes	No	No	No	Yes	Yes	Yes	No	No
Shinoda et al., 2020 [46]	Yes	Yes	Yes	Yes	No	No	Yes	Yes	Yes	Yes
Koetsier et al., 2020 [11]	No	No	No	No	Yes	No	No	No	No	No
Vallejo et al., 2020 [47]	Yes	Yes	No	Yes	Yes	Yes	No	Yes	Yes	No
Liao et al., 2020 [45]	Yes	Yes	No	Yes	Yes	Yes	No	Yes	Yes	Yes
Gilbert et al., 2018 [26]	No	No	No	No	Yes	Yes	No	No	Yes	No
Sun et al., 2017 [12]	Yes	Yes	Yes	Yes	Yes	Yes	Yes	Yes	No	No
Crosby et al., 2015 [13]	No	Yes	No	Yes	Yes	Yes	No	No	No	No
Zhang et al., 2015 [14]	Yes	Yes	No	Yes	Yes	No	No	Yes	Yes	No
Sato et al., 2014 [49]	Yes	Yes	No	No	Yes	Yes	No	Yes	Yes	No
Zhang et al., 2014 [15]	Yes	Yes	No	Yes	No	Yes	No	No	No	Yes
Yang et al., 2014 [28]	Yes	Yes	No	Yes	Yes	Yes	No	Yes	Yes	No
Yuan et al., 2014 [50]	Yes	Yes	No	No	No	Yes	No	Yes	Yes	No
Jongen et al., 2014 [48]	Yes	Yes	No	Yes	Yes	No	Yes	Yes	No	No
Song et al., 2014 [27]	Yes	Yes	No	No	Yes	Yes	Yes	Yes	Yes	Yes
Sato et al., 2013 [34]	Yes	Yes	No	Yes	No	No	Yes	Yes	Yes	No
Ultenius et al., 2013 [16]	Yes	Yes	No	Yes	Yes	Yes	No	Yes	No	Yes
Janssen et al., 2012 [17]	Yes	Yes	No	Yes	Yes	Yes	Yes	Yes	Yes	Yes
Smits et al., 2012 [29]	No	No	No	No	Yes	No	No	Yes	No	No
Song et al., 2011 [35]	Yes	Yes	No	No	No	Yes	Yes	Yes	No	No
Yang et al., 2011 [30]	Yes	Yes	Yes	Yes	No	No	No	Yes	Yes	No
Smits et al., 2009 [18]	Yes	Yes	No	Yes	No	No	Yes	Yes	Yes	No
Song et al., 2009 [36]	Yes	Yes	No	Yes	No	No	Yes	Yes	Yes	No
Schechtmann et al., 2008 [37]	No	No	No	No	Yes	No	No	Yes	Yes	No
Wallin et al., 2003 [31]	Yes	Yes	No	No	No	Yes	Yes	No	No	No
Yakhnitsa et al., 1999 [32]	No	No	No	No	No	No	No	Yes	No	No
Cui et al., 1998 [19]	Yes	Yes	No	Yes	Yes	Yes	No	Yes	No	No
Cui et al., 1997 [20]	Yes	Yes	No	No	No	Yes	Yes	Yes	No	No
Stiller et al., 1996 [22]	Yes	Yes	No	Yes	Yes	Yes	Yes	Yes	Yes	No
Cui et al., 1996 [21]	Yes	Yes	No	Yes	Yes	Yes	No	Yes	No	No
Linderoth et al., 1994 [23]	Yes	Yes	Yes	Yes	No	No	Yes	Yes	No	No

The studies were evaluated for bias based on key criteria including sequence generation, baseline characteristics, allocation concealment, random housing, blinding (performance and detection bias), random outcome assessment, incomplete outcome data, selective outcome reporting, and other sources of bias.

Discussion

Our systematic review consolidates preclinical evidence regarding the cellular and physiological mechanisms by which SCS alleviates pain in animal models. The experimental paradigms employed are classified as either neuropathic or inflammatory pain models, which exhibit clinical relevance to the phenomenon of chronic pain. The majority of investigations utilized rats in their animal study with the exception of one study that fails to specify the species employed.

While the SYRCLE tool does not generate a numerical summary score, the number of "Yes" responses per study ranged from one to nine, with an approximate average of 6.09. This indicates a moderate and inconsistent risk of bias across studies and underscores the importance of interpreting preclinical findings with caution. Studies are categorized into three mechanistic domains (inhibition of ascending nociceptive transmission at the dorsal horn, enhancement of descending inhibition and neuroimmune modulation: microglial and astrocytic effects) based on established literature precedence, detailed experimental methodologies, and the mechanistic conclusions drawn in each study. Inhibition of ascending nociceptive transmission at the dorsal horn was further subdivided into mechanisms at the molecular level and electroneurophysiological studies.

Inhibition of Ascending Nociceptive Transmission at the Dorsal Horn

Molecular biology studies: SCS inhibits ascending nociceptive signaling at the dorsal horn through both molecular and electroneurophysiological mechanisms. On the molecular level, SCS enhances inhibitory neurotransmission, particularly via GABAergic and endocannabinoid pathways, while also reducing excitatory drive in dorsal horn neurons. Multiple studies report increased GAD65 expression and enhanced GABA release following stimulation [16,22]. GABA(B) receptor activation plays a central role in suppressing excitatory amino acid release, an effect reversed by GABA(B) antagonists [20-21]. Moreover, SCS engages adenosine receptor pathways to normalize tactile thresholds [19], and chronic pain conditions such as diabetic neuropathy may limit SCS efficacy due to reduced KCC2 expression and impaired GABA(A)-mediated inhibition [17]. Additionally, CB1 receptor-mediated endocannabinoid activation contributes to long-lasting analgesic effects following repetitive SCS [12]. The impact of SCS on wide-dynamic range neurons appears to be frequency-sensitive, with modeling studies showing GABAergic tone shaping optimal parameters [15], and projection neuron modulation extending beyond classical gate control mechanisms [14]. Notably, dorsal root ganglion stimulation appears to provide analgesia through non-GABAergic pathways, emphasizing mechanistic diversity [11].

Electroneurophysiological studies: Electroneurophysiological studies further support these findings by demonstrating circuit-level modulation of spinal neurons. SCS alters firing patterns in the dorsal horn in a frequency and duration-dependent manner, favoring the activation of inhibitory interneurons [25]. Markers of neuronal activation, such as c-Fos expression, are elevated after stimulation confirming both early and late-phase neural engagement [18]. Evoked compound action potential (ECAP) and electromyography (EMG) recordings during lateralized stimulation demonstrate preferential ipsilateral recruitment of spinal neurons [24]. Moreover, SCS reduces the long-term potentiation of C-fiber inputs [31], thereby modulating central sensitization. Intensity-dependent inhibition of wide-dynamic range neurons further supports the idea that optimal stimulation parameters are critical for efficacy [28].

Additionally, low-frequency sub-perception SCS activates only a limited set of dorsal column axons that engage dorsal horn inhibitory circuits without recruiting ascending pathways [26]. It was also demonstrated that low-threshold A-fiber activation at sub-motor intensities contributes significantly to analgesia by selectively engaging local spinal circuits [30]. Finally, suppression of dorsal horn hyperexcitability and principal after discharges by SCS underscores its potent local inhibitory action [32].

Enhancement of Descending Inhibition

SCS enhances descending inhibitory control by promoting the spinal release of key neuromodulators, including serotonin, endogenous opioids, and acetylcholine. Activation of spinal 5-HTt and 5-HT receptors facilitates GABAergic inhibition [36], while frequency-dependent release of opioid peptides such as methionine enkephalin contributes to analgesia blocked by antagonists [33-34]. Cholinergic signaling via muscarinic M4 receptors further supports nociceptive modulation [37], illustrating the multifaceted chemical basis of SCS-induced descending inhibition.

Neuroimmune Modulation: Microglial and Astrocytic Effects

Chronic pain is increasingly understood as a neuroimmune condition involving sustained activation of microglia and astrocytes within the spinal cord. SCS counters this by downregulating pro-inflammatory mediators such as IL-1β, TNF-α, NF-κB, and TLR4 [40,50], reducing CSF1 signaling [41], and inhibiting the p38 MAPK and MAPK/ERK pathways [44-45]. It also suppresses superficial microglial activation in key dorsal horn segments [46] and shifts glial states toward a neuroprotective phenotype using advanced waveform-specific paradigms such as DTMP (differential target multiplexed programming) [42] and HF10 SCS (10-kilohertz high-frequency spinal cord stimulation) [38]. Proteomic and transcriptomic studies demonstrate that these effects extend to gene expression regulation and oxidative stress pathways [39,43], while acute imaging studies confirm rapid reductions in spinal metabolic activity following stimulation [48]. These findings support the rationale for waveform-specific programming in clinical practice, particularly for patients with neuroinflammatory phenotypes, and may inform future device designs that target glial modulation more precisely.

## Conclusions

While extensive research has been conducted on SCS in animal models, a systematic review specifically focusing on the pain mechanisms modulated by SCS in spinal cord cells has been lacking. The mechanisms underpinning SCS analgesia, as delineated in the comprehensive analysis of selected studies, are categorized into three primary domains: the inhibition of ascending nociceptive transmission at the dorsal horn, the enhancement of descending inhibition, and neuroimmune modulation, specifically focusing on the effects of microglia and astrocytes. The experimental paradigms employed consist of either neuropathic or inflammatory pain models in rats, which bear significant clinical correlations to chronic pain phenomena. This review addresses that gap by providing a comprehensive analysis of the available literature, categorizing cellular pain modulation mechanisms, assessing study quality, and outlining key trends to guide translational and therapeutic advancements. Nevertheless, the therapeutic implications drawn from this review should be interpreted with caution given the exclusive use of animal data and the variability in methodological quality across studies.

## Appendices

**Table 5:** Full search strategy utilizing Cochrane controlled trials register

ID	Search
#1	MeSH descriptor: [Spinal Cord Stimulation] explode all trees
#2	MeSH descriptor: [Electric Stimulation] explode all trees
#3	MeSH descriptor: [Electric Stimulation Therapy] this term only
#4	((spine or spinal or transspinal or "trans spinal" or column* or epidural or lumbar or "dorsal column" or sacral or coccygeal or "conus medullari" or "conus medullaris" or "conus terminali" or "conus terminalis" or "medulla spinali" or "medulla spinalis" or myelon* or "thoracic cord" or "thoracic cords") NEAR/3 stimulat*)
#5	spinal cord stimulation or "spinal cord stimulations"
#6	(stimulat* NEAR/1 frequency)
#7	((therapy or therapies) NEAR/1 frequency)
#8	(burst NEAR/1 stimulat*)
#9	MeSH descriptor: [Electrodes, Implanted] explode all trees
#10	(electrode* NEAR/1 implant*)
#11	neuromodulat*
#12	#1 OR #2 OR #3 OR #4 OR #5 OR #6 OR #7 OR #8 OR #9 OR #10 OR #11
#13	MeSH descriptor: [Pain] explode all trees
#14	MeSH descriptor: [Pain Management] explode all trees
#15	(pain or pains):ti,ab,kw
#16	MeSH descriptor: [Nociceptors] explode all trees
#17	(nocicept*):ti,ab,kw
#18	#13 OR #14 OR #15 OR #16 OR #17
#19	#12 AND #18
#20	mechanis*
#21	#19 AND #20
#22	MeSH descriptor: [Models, Animal] explode all trees
#23	(animal* or rodent* or rat or rats or mouse or mice or "guinea pig" or "guinea pigs" or hamster* or rabbit* or cat or cats or dog or dogs or cattle or cow or cows or horse or horses or donkey* or mule or mules or monkey* or "nonhuman primate" or "non-human primate" or "nonhuman primates" or "non-human primates" or chimpanzee* or sheep or swine or pig or pigs or chicken or chick or chicks):ti,ab,kw
#24	#22 OR #23
#25	#21 AND #24
